# Updated clinical recommendations for the use of tibolone in Asian women

**DOI:** 10.3109/13697131003681458

**Published:** 2010-05-06

**Authors:** K-E. Huang, R. Baber

**Affiliations:** Center for Menopause and Reproductive Medicine Research and Department of Obstetrics and Gynecology, Chang Gung Memorial Hospital-Kaohsiung Medical Center, Kaohsiung, Taiwan; * Sydney Medical School, The University of Sydney, Royal North Shore Hospital and North Shore Private Hospital, St. Leonards, Australia

**Keywords:** TIBOLONE, POSTMENOPAUSAL, ASIAN, TREATMENT ALGORITHM, REVIEW

## Abstract

Tibolone, which is indicated for the relief of climacteric symptoms and the prevention of osteoporosis in postmenopausal women, has a tissue-specific mode of action different to that of conventional hormone replacement therapy (HRT). A large proportion of Asian postmenopausal women experience symptoms that most frequently include musculoskeletal pain, insomnia, forgetfulness, hot flushes and sexual dysfunction, and there is a need to address their specific requirements. Recent studies show that, in comparison to HRT, tibolone is as effective in alleviating menopausal symptoms and preventing bone loss, has a greater positive effect on sexual dysfunction and is associated with less vaginal bleeding, but it is rarely mentioned in guidelines for menopausal treatment. Levels of awareness amongst women about treatments for menopausal symptoms vary between Asian countries but, even in countries where awareness is high, HRT usage is much lower than in the West. To provide a practical approach to the use of tibolone in Asian postmenopausal women, a panel of experts in the management of menopause from 11 Asia Pacific countries has developed recommendations for its use, based on the evidence from clinical studies published since 2005. However, as much of the clinical data reviewed are from international studies, the recommendations and the treatment algorithm presented here are widely applicable.

## INTRODUCTION

Tibolone is indicated for the relief of climacteric symptoms and the prevention of osteoporosis in postmenopausal women. It is described as a selective tissue estrogenic activity regulator (STEAR) because it has specific effects in different tissues after conversion to three active metabolites following oral ingestion^1^. Estrogenic metabolites act centrally, on bone and the vagina, and, together with androgenic metabolites, relieve hot flushes and improve energy and sexual well-being. The progestogenic D4-isomer of tibolone prevents stimulation of the endometrium, while breast tissue stimulation is minimized due to the effects of tibolone on local enzyme activity, which inhibit conversion of endogenous estrone sulfate to the active hormones estrone and 17β-estradiol^2^.

Tibolone and conventional hormone replacement therapy (HRT, either estrogen therapy (ET) or estrogen-progestogen therapy (EPT)) are considered to offer equivalent relief of vasomotor symptoms of menopause^3^–^5^, but there is evidence that tibolone results in better female sexual function^6^–^8^, particularly with respect to desire and arousal^9^, probably due to its combined estrogenic and androgenic properties. Improvements on psychological and somatic scales have also been found to be superior with tibolone compared with traditional HRT^10^. Tibolone is well tolerated^11^ and does not cause weight gain in menopausal women. Tibolone does not stimulate the endometrium^13^,^14^, and, in comparison to EPT, does not increase the size of uterine myomas^15^. The Long-term Intervention on Fractures with Tibolone (LIFT) study showed that tibolone reduces the risk of vertebral fractures and possibly colon cancer, but increased the risk of stroke in older women with osteoporosis^16^. Tibolone does not increase mammographic density^17^ and, in the LIFT study in older women with no previous history, tibolone reduced the risk of breast cancer^16^. However, the Livial1 Intervention Following Breast Cancer; Efficacy, Recurrence and Tolerability End-points (LIBERATE) trial showed an increased risk of recurrence of breast cancer with tibolone^18^, and hence it is contraindicated in women with known, past or suspected breast cancer.

## RATIONALE FOR UPDATED RECOMMENDATIONS FOR TIBOLONE

It was once assumed that the menopause was an event whose timing and physiological implications were universally the same. However, both biological and cultural variables can influence symptomatology. Age, symptoms and mortality can vary tremendously between different parts of the world, even within countries in Asia Pacific^19^,^20^ (discussed further below). Discrepancies between physicians’ perceptions and women's reports about the reasons for which they consult physicians at menopause are also seen, and prescription patterns and perceived benefits of HRT can reflect local medical culture.

The value of addressing region-specific elements of menopause care and decision-making has been recognized by the Asia Pacific Menopause Federation (APMF) and their 2008 consensus statement highlights the essential nature of individualized management^21^. Because tibolone has a different pharmacological profile to conventional HRT, separate guidelines that provide a practical tool for everyday use by gynecologists and general practitioners are of value. However, there is no mention of tibolone in the European Menopause and Andropause Society (EMAS) 2004/ 2005 position statements on peri- and postmenopausal HRT^22^, the 2007 International Menopause Society (IMS) updated recommendations on postmenopausal hormone therapy^23^, or the 2008 position statement of the North American Menopause Society^24^. Guidelines generated at the fourth Amsterdam Menopause Symposium in 2004^22^ stated only that ‘Tibolone is as effective as hormone therapy (HT) in treating symptoms and preventing bone loss, and it improves sexuality'. Although Kenemans and colleagues^25^ published an excellent summary of the evidence-based consensus findings for the use of tibolone in 2005, that paper did not contain detailed practical guidelines for its use and several large, randomized trials have since yielded additional data on tibolone's efficacy and safety profile.

A panel of experts in the management of the menopause from 11 Asia Pacific countries (Australia, China, India, Indonesia, Korea, Malaysia, the Philippines, Singapore, Taiwan, Thailand and Vietnam) therefore met in Siem Reap, Cambodia, in June 2009 to develop recommendations for the use of tibolone to treat climacteric symptoms in postmenopausal Asian women.

## EXPERIENCE OF MENOPAUSE IN ASIAN WOMEN

Numerous reviews and studies show that the relative prevalence of postmenopausal symptoms in Asian women can differ substantially from those in Western women and between (and even within) Asian countries^26^-^30^.

Whereas vasomotor symptoms such as hot flushes and night sweats are generally the most commonly reported menopausal symptoms in Western countries^20^,^31^, this is not always the case in many Asian countries. In a 2006 survey of over 1000 Asian women, the most common reason for seeking treatment was insomnia (reported by 42%). Hot flushes were reported by 37% of the Asians who sought treatment, compared to 59% of Europeans in a separate survey^32^. In the Pan Asian Menopause (PAM) study of 1028 Asian women from nine ethnic groups^27^, the prevalence of vasomotor symptoms ranged from 5% in Indonesian women to 100% in Vietnamese women. The prevalence of vasomotor symptoms also varies within countries: a study of menopausal symptoms in Chinese women showed a significantly lower prevalence among rural farming women (28%) than among urban women (47%)^29^, and the prevalence of hot flushes varied from 25% to 80% in Thai postmenopausal women in three different studies^31^. Amongst Australian postmenopausal women, the prevalence of vasomotor symptoms ranged from 45% to 80% over five studies^31^.

In the PAM study, body or joint aches were the most commonly reported symptoms (by 88% overall - the range was from 76% in Korean women to *96%* in Vietnamese women)^27^. Bone or joint pain was also the most prevalent currently experienced symptom (by 38% of participants) in a Korean study of 1201 Korean menopausal and postmenopausal women^30^, followed by forgetfulness (35%). Forgetfulness was the second most common symptom experienced overall in that study (by 48%) and in the PAM study (by 81%). Other commonly reported symptoms (by over 45% of postmenopausal women in different Asian countries) include insomnia, headache, irritability and palpitations 19,27,28,30.

Vaginal dryness or irritation was reported by 55% of women in the PAM study and dyspareunia by 30%^27^. The prevalence of dyspareunia was 44% in a Malaysian study of postmenopausal women^33^, 32% in a Taiwanese study^28^ and ranged from 7.7% in Singaporeans to 46.9% of Indonesians in a study of menopausal symptoms across seven Asian countries^19^.

## REVIEW OF MOST RECENT DATA FROM INTERNATIONAL STUDIES OF TIBOLONE

Since the 2005 publication of consensus recommendations for the use of tibolone as postmenopausal therapy 5, data from seven large, randomized, clinical trials of tibolone have been published and are summarized in the Appendix. These studies confirmed that tibolone is significantly more effective than placebo^18^ and as effective as low-dose continuous combined estradiol plus norethisterone acetate (E2/NETA)^34^ in reducing vasomotor symptoms.

Tibolone was associated with significantly greater improvements in sexual interest than transdermal E2/ NETA, and showed a trend towards greater improvement in sexual function^8^. Incidences of both vaginal bleeding and breast tenderness/pain were significantly lower with tibolone than with either oral or transdermal E2/NETA^8^,^34^ or continuous combined conjugated equine estrogen plus medroxyprogesterone acetate (CEE/MPA)^35^. Data from the Tibolone Histology of the Endometrium and Breast Endpoints Study (THEBES) confirmed the endometrial safety of tibolone over 2 years at doses of both 1.25 mg and 2.5 mg per day with no cases of endometrial hyperplasia or cancer in either dosage group^35^.

Tibolone (1.25 and 2.5 mg, respectively) increased lumbar and hip bone mineral density to a significantly greater extent than placebo in women with16 and without osteoporosis^18^, as did a dose of 1.25 mg/day compared with raloxifene in a study of older osteopenic women (mean age *66* years)^36^. The lower dose also reduced the risk of vertebral and non-vertebral fractures in older osteoporotic women (mean age 68.3 years) in the LIFT study^16^.

A greater risk of stroke, which increased further with age, was observed with tibolone than with placebo in the LIFT study^16^. However, the differences in absolute risk between treatment groups were not statistically significant, the study population was elderly (60-85 years) and no increase in the risk of venous thromboembolism was seen. By contrast, no increased risk of stroke was observed in the THEBES study (mean age 54.4 years) or in a case-control study of women aged 50-79 years (mean 70.3 years)^37^, although the number of tibolone-treated cases in the latter study was small. The LIFT investigators concluded that tibolone should not be used in elderly women (i.e. over 60 years) or those who have strong risk factors for stroke, such as hypertension, smoking, diabetes and atrial fibrillation^16^.

In the Osteoporosis Prevention and Arterial effects of tiboLone (OPAL) study, which was designed to compare the risk of cardiovascular disease between tibolone, CEE/MPA and placebo, tibolone reduced total cholesterol and high density lipoprotein (HDL) cholesterol to a greater extent than placebo, whereas CEE/ MPA increased HDL cholesterol^38^. The mean increase in carotid intima-media thickness (CIMT), which is related to risk of cardiovascular disease, was similar with tibolone and CEE/MPA and greater than with placebo. However, inconsistencies in the CIMT findings were observed between the European and US cohorts in this study^39^. It was concluded that neither treatment showed either beneficial or harmful effects with regard to atherosclerosis^38^,^39^. No increased risk of myocardial infarction was found with tibolone in a 5-year national cohort study in Denmark^40^.

In the LIFT study, compared with placebo, tibolone was associated with a reduced risk of breast cancer in older women with no prior history of the disease^16^. Observational studies have provided conflicting findings on this issue: a large UK case-control study showed no increase in breast cancer risk^41^, while the Million Women Study showed an increased risk with tibolone^42^. The LIBERATE trial was conducted to determine whether tibolone could be prescribed to women with a previous history of breast cancer to alleviate their menopausal symptoms without increasing their risk of recurrence. However, this study showed a significantly greater risk of breast cancer recurrence in the tibolone group than in the placebo group, despite the fact that the majority of the study population was receiving adjuvant systemic therapy. At study entry, *66%* of the study populations were receiving tamoxifen and 6% aromatase inhibitors. Use of aromatase inhibitors increased during the trial and, at the study close, approximately 80% were on adjuvant endocrine therapy^18^. Subgroup analyses suggested that the interference of tibolone in users of aromatase inhibitors was more severe than in tamoxifen users, where the activation of the estrogen receptor by the estrogenic metabolites of tibolone is prevented by high-affinity hydroxyl-tamoxifen molecules. The investigators concluded that the discrepancies between these findings and those of the LIFT study arose because the two study populations differed in many respects, including hormonal risk factors for breast cancer, and because the effects of tibolone on healthy breast tissue most probably differ from those on cancer cells as tibolone may exert an estrogenic effect on occult, dormant breast cancer metastasis. On the basis of data from the LIBERATE trial, tibolone is contraindicated for women with known, past or suspected breast cancer.

## CLINICAL EXPERIENCE OF TIBOLONE IN ASIAN WOMEN

Several randomized clinical studies of tibolone in Asian populations have been published, and, although these are small (generally fewer than 100 participants), the findings are consistent with those of the larger international trials described previously. A 6-month study of tibolone (2.5 mg/day) versus CEE/MPA (0.625/5 mg/day) in Taiwanese women showed that both treatments preserved cognitive function as assessed by the Cognitive Abilities Screening Instrument and the Mini Mental State Examination^43^. In another Taiwanese study of similar design, tibolone resulted in significantly higher scores than CEE/MPA for all aspects of sexuality, as assessed by the McCoy sex scale, including vaginal dryness and painful intercourse^44^. A third Taiwanese study showed a significant increase in lumbar bone mineral density with tibolone (but not CEE/MPA) and that tibolone alleviated climacteric complaints (as assessed using the Greene Climacteric Scale (GCS)) more quickly and effectively than CEE/MPA^45^. In all three studies, no changes in endometrial thickness were observed with either treatment, and tibolone use was associated with a substantially lower incidence of vaginal bleeding compared with CEE/MPA. A Hong Kong study of the same design also showed a significantly beneficial effect with tibolone on GCS somatic sub-scores^46^.

Reduced levels of total cholesterol, triglycerides and HDL cholesterol were seen with tibolone in the third Taiwanese study, although low density lipoprotein (LDL) cholesterol levels increased slightly. Similar findings were reported in a Korean study 7, although, in the latter, LDL cholesterol levels were slightly reduced with tibolone. In the Korean study, tibolone also significantly improved flow-mediated brachial artery dilator response to the same extent as CEE/ MPA, but did not significantly change high-sensitivity Creactive protein or antithrombin III levels. The investigators concluded that, overall, tibolone has complex effects on lipids, some that might be expected to improve the cardiovascular risk profile and others that might worsen it.

The findings of increased risk of stroke with tibolone in the LIFT study may not have as strong implications for Asian women as for Western women. The incidence of stroke is generally lower in Asian than Western women^48^,^49^, and obesity and thrombophilia, two of the largest risk factors, tend to be less prevalent in Asian populations^50^.

The breast tissue of Asian women tends to show greater mammographic density than that of Western women^51^,^52^, which can impair the interpretation of mammograms. HRT has been shown to cause an increase in mammographic density^17^,^53^, but tibolone has been shown to either decrease^53^ or have no effect on mammographic density^17^,^54^. Hence, tibolone treatment may be preferable to HRT in symptomatic menopausal women with mammographically dense breast tissue.

## RECOMMENDATIONS FOR TIBOLONE USE

Based on the evidence described here, the expert panel developed the consensus statements for the use of tibolone that are shown in Table 1. The levels of evidence for each statement were assigned using the criteria established by the Oxford Centre for Evidence-based Medicine^55^.

**Table 1:** Consensus statements on the use of tibolone and levels of supporting evidence

Updated statements and/or new evidence published since 2005	Level of evidence*
Tibolone is as effective as currently used EPT/ET regimens in the management of climacteric symptoms^34^	1b
Tibolone treats vaginal atrophy and alleviates local vaginal symptoms^34^	1b
Tibolone has a positive effect on sexual well-being and is more effective than oral EPT/ET in some respects, namely arousal, desire, and satisfaction^8^,^34^	1b
Tibolone positively affects mood and quality of life^8^,^34^	1b
Tibolone prevents bone loss and is as effective as standard doses of EPT/ET and more effective than raloxifene 36	1b
Tibolone reduces the risk of vertebral and non-vertebral fracture in older osteoporotic women. The absolute reduction was greater among women who had already had a vertebral fracture than among those who had not^16^	1b
Tibolone does not stimulate the endometrium or induce endometrial hyperplasia or carcinoma in postmenopausal women in randomized controlled clinical trials and has a low incidence of bleeding^34^,^35^,^61^	1b
In observational studies, an increased relative risk of endometrial cancer has been shown^56^,^57^	3b
Tibolone causes less breast tenderness and less mastalgia than EPT^34^	1b
Tibolone does not increase mammographic density	2b
Tibolone, taken by women with a personal history of breast cancer, is associated with an increased risk of recurrence 18	1b
The evidence of tibolone use and increased risk of breast cancer from observational studies remains inconclusive^41^	3b
Tibolone 1.25 mg does not increase breast cancer risk in older osteoporotic women with no history of breast cancer^16^	1b
There are still no hard endpoint data on the effect of tibolone on cardiovascular health^38^	1b
Tibolone has different effects on lipids compared with EPT/ET^38^	1b
Tibolone increases CIMT in a manner similar to EPT^38^	1b
In one randomized, controlled trial, use of tibolone 1.25 mg in older women was associated with an increased risk of stroke^16^. Hence, tibolone should be used with caution in elderly women (i.e. over 60 years) and should not be used in those who have strong risk factors for stroke	1b
Tibolone did not increase the risk of stroke, VTE or myocardial infarction in observational studies^37^,^40^	2b

* Definitions of levels of evidence: 1b, individual randomized trials; 2b, individual cohort study; 3b, individual case-control study^55^ EPT, estrogen-progestogen therapy; ET, estrogen therapy; CIMT, carotid intima-media thickness; VTE, venous thromboembolism

The panel agreed that there was a need for data on the possible effects of tibolone on musculoskeletal symptoms, given their frequency in Asian women, as well as for more long-term data with regard to cardiovascular endpoints and endometrial cancer in these populations. Although randomized, controlled trials have shown no increase in hyperplasia or cancer with tibolone over a mean duration of 2 years, two observational studies have reported an increased risk of endometrial cancer in tibolone users^56^,^57^. However, it is not known whether this result is biased due to preexisting endometrial abnormalities or prior use of other forms of HRT.

Published data on the possible effects of tibolone in diabetes and on stress incontinence in Asian women are also lacking, as are large studies of the effect of tibolone on breast cancer risk in these populations. It was also suggested that the efficacy of lower doses than those recommended in the prescribing information might be explored in Asian women via controlled studies.

## ALGORITHM FOR THE USE OF TIBOLONE IN ASIAN MENOPAUSAL WOMEN

**Figure 1:**
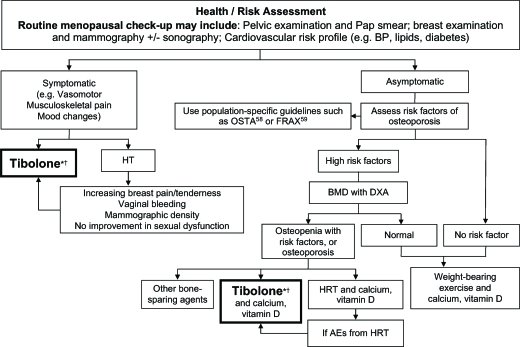
Algorithm for the use of tibolone in Asian menopausal women. *, Tibolone treatment is preferable to hormone replacement therapy (HRT) for postmenopausal women who report mastalgia, breast tenderness, increasing mammographic density and sexual problems. †, Contraindications for tibolone should be considered the same as for estrogen-progestogen therapy/estrogen therapy (EPT/ET). In addition, tibolone should only be used in women with no history of breast cancer. Tibolone should be used with caution in elderly women (i.e. over 60 years) and should not be used in those who have strong risk factors for stroke. OSTA, Osteoporosis self-assessment tool for Asians; FRAX, World Health Organization's fracture risk assessment tool; BP, blood pressure; BMD, bone mineral density; DXA, dual-energy X-ray absorptiometry; AEs, adverse events

To provide clear guidance on when tibolone can be used and how it fits in with other available treatments for menopausal symptoms, the algorithm shown in Figure 1 was developed. Although symptoms and treatment modalities particular to Asian postmenopausal women are taken into account, the algorithm is generally applicable to women of all ethnicities.

During the discussions that resulted in this algorithm, the expert panel agreed that, on the basis of the clinical evidence available, women who might benefit from a switch to tibolone from HRT included those who experienced:

1. An increase in breast pain despite HRT dose adjustment;
2. Increased breast density that resulted in an unreadable mammogram;
3. Low libido;
4. Mood disorders; and
5. Persistent bleeding problems (providing no histopathological reasons for this exist).

Asian women can be more reticent about discussing sexual dysfunction than Western women, despite the fact that it is reported by up to 50% of postmenopausal women in some Asian countries^19^,^27^,^60^. Nevertheless, in a multinational survey of 1000 Asian postmenopausal women, 71% regarded sex as an important part of marriage^20^, and, hence, the inclusion of this item in the algorithm is intended to prompt the clinician to overcome any reluctance to raise the subject with postmenopausal patients. As tibolone is more effective in improving sexual function than HRT^8^,^34^,^44^, women who experience related problems are likely to benefit from it.

Contraindications for tibolone should be considered the same as for EPT/ET. As mentioned previously, tibolone should only be used in women with no history of breast cancer. Although there are no data to suggest an increased risk of venous thromboembolism to date, on the basis of findings in the LIFT study^16^, tibolone should be used with caution in elderly women (i.e. over 60 years) or those who have strong risk factors for stroke such as hypertension, smoking, diabetes and atrial fibrillation.

## CONCLUDING REMARKS

Asians are multiethnic and differ with regard to dietary habits, attitudes to the menopause and menopausal symptoms, both amongst themselves and from Western women. This paper is intended to address the need for clear, up-to-date recommendations for the use of tibolone, particularly in light of these differences. Although data from large clinical trials on the effects of tibolone in Asian women are limited, we have reviewed data on menopausal symptoms in Asian women and identified areas in which the specific use of tibolone might offer particular advantages to them as they pass the menopause transition. Based on a thorough review of the most recent clinical trial data on tibolone, its known effects have been summarized in consensus statements that are supported by levels of evidence, and a treatment algorithm is provided to aid in the selection of appropriate treatment for all postmenopausal women.

## ASIA PACIFIC TIBOLONE CONSENSUS GROUP

Professor Ko-En Huang, Center for Menopause and Reproductive Medicine Research and Department of Obstetrics and Gynecology, Chang Gung Memorial Hospital-Kaohsiung Medical Center, Kaohsiung, Taiwan, ROC; Professor Byung-Koo Yoon, Department of Obstetrics and Gynecology, Samsung Medical Centre, Sungkyunkwan University School of Medicine, Seoul, Korea; Professor Alastair MacLennan, Discipline of Obstetrics and Gynecology, The University of Adelaide, Australia; Professor Xin Yang, Department of Obstetrics and Gynecology, Peking University First Hospital, Beijing, China; Professor Qinjie Tian, Department of Obstetrics and Gynecology, Peking Union Medical College Hospital, Beijing, China; Dr Lilia Luna, Department of Obstetrics and Gynecology, Capitol Medical Center, Manila, Philippines; Dr Hean Choon Ong, Klinik Wanita, Kuala Lumpur Menopause Center, Kuala Lumpur, Malaysia; Associate Professor Rod Baber, Sydney Medical School, The University of Sydney, Royal North Shore Hospital and North Shore Private Hospital, St Leonards, Australia; Professor Heung Yeol Kim, Department of Obstetrics and Gynecology, School of Medicine, Kosin University, Pusan, Korea; Dr Ratnabali Chakravorty, Department of Gynecology and Obstetrics, MGM Medical College, Kishanganj, India; Professor Nimit Taechak-raichana, Department of Obstetrics and Gynecology, Chulalongkorn Medical School, Bangkok, Thailand; Professor Tran Thi Loi, Department of Obstetrics and Gynecology, HCMC Medicine and Pharmacy University, Ho Chi Minh City, Vietnam; Dr Su Ling Yu, Department of Obstetrics and Gynecology, Singapore General Hospital, Singapore; Professor Dr med. Ali Baziad, Division of Reproductive Immunoendocrinology, Cipto Mangunkusumo General Hospital, Jakarta, Indonesia.

## Appendix

**Appendix 1:** Summary of data from large international clinical studies of tibolone published since 2005

	Long-Term Intervention on Fractures with Tibolone (LIFT)^16^	Tibolone Histology of the Endometrium and Breast Endpoints Study (THEBES)^35^	Livial® Intervention following Breast cancer: Efficacy, Recurrence and Tolerability Endpoints (LIBERATE)^18^
Design	Randomized, placebo-controlled study of older postmenopausal women (60–85 years) with hip and/or spine BMD *T* score of ≤ ← 2.5, or ≤ ← 2.0 with vertebral fracture	Double-blind study of postmenopausal women randomized (1 : 1 : 2) to receive different doses of tibolone or CEE + MPA	Randomized, double-blind, placebo-controlled study to assess safety and effi cacy of tibolone in women with vasomotor symptoms and history of breast cancer
*n*	4538	3240	3098
*Mean age* (years)	68.3	54.4	52.7
Dose	Tibolone 1.25 mg/day vs. placebo	Tibolone 1.25 mg/day vs. tibolone 2.5 mg/day vs. CEE/MPA 0.625/2.5 mg/day	Tibolone 2.5 mg/day vs. placebo
Primary endpoint	Reduction in incidence of new vertebral fractures after 3 years	Endometrial safety (hyperplasia, cancer) of tibolone (1.25 and 2.5 mg/day) after 1 and 2 years	To demonstrate the non-inferiority of tibolone compared with placebo regarding breast cancer recurrence
Major findings	*Fractures*	*Endometrium*	*Breast cancer recurrence*
	Tibolone reduced the risk of both vertebral (45% compared with placebo) and non-vertebral (26% compared with placebo) fractures	No incidence of endometrial hyperplasia or cancer with tibolone 1.25 or 2.5 mg; two cases of endometrial hyperplasia observed in CEE/MPA group; no endometrial carcinoma in any group	Overall, increased risk of recurrence with tibolone compared with placebo: 15.2% recurrence with tibolone vs. 10.7% with placebo (*p* = 0.001).Difference in incidence was not immediate, but emerged after 1 year. Most pronounced difference was observed for distal recurrence: 11.0% with tibolone vs. 7.8% with placebo (*p* = 0.007)
	Tibolone showed a 6.6% increase in lumbar spine BMD compared to 1.4% with placebo at Year 4 (*p* < 0.001)	Small mean increase in double-wall endometrial thickness by TVUS in both tibolone and CEE/MPA over 2 years but no difference between groups	
	Total hip BMD increased by 2.8% with tibolone by Year 4, but no change was seen with placebo (*p* < 0.001)	Both treatments considered safe with regard to endometrium at study end	
	*Stroke*	*Vaginal bleeding/spotting*	*Hot flushes*
	Increased risk of stroke within 1 year compared with placebo, with a relative hazard of 2.19 by 4 years (95% CI 1.4–4.23); *p* = 0.02]	Over 2 years, vaginal bleeding occurred in 13.3%, 20.2% and 42.6% of those in tibolone 1.25 mg, 2.5 mg and CEE/MPA groups, respectively	Significantly greater decrease from baseline with tibolone than placebo at all time points (*p* < 0.0001)
	*Cancer*	*Breast pain*	*BMD*
	Decreased risk of invasive breast cancer with tibolone compared with placebo (relative hazard 0.32; 95% CI 0.13–0.80; *p* = 0.02)	4.3% in tibolone groups (combined) vs. 12.7% with CEE/MPA (*p* < 0.001)	Significantly greater increases with tibolone than placebo from baseline in lumbar (3.3%) and hip BMD (2.9%) (*p* < 0.0001)
	Decreased risk of colon cancer with tibolone compared with placebo (relative hazard 0.31; 95% CI 0.10–0.96; *p* = 0.04)			
Notes	Study stopped prematurely after a mean follow-up period of 2.9 years as endpoints had been reached and stroke increased		Discontinued just before 3 years' exposure as unlikely to meet pre-specified inferiority criteria

	Trial to compare the effects of tibolone (Livial®) and continuous combined low-dose E2/NETA (Activelle®) (TOTAL)^34^	Osteoporosis Prevention and Arterial effects of tiboLone (OPAL)^38^	Livial® International Study in sexual Arousal disorders (LISA)^8^,^61^	Study of Tibolone's Effects on osteoPenia (STEP)^36^,^62^
Design	Multinational, randomized, double-blind study of tibolone vs. low-dose continuous combined E2/NETA	Multicenter, randomized controlled trial of tibolone vs. CEE/MPA or placebo	Randomized, controlled trial comparing tibolone with continuous combined transdermal E2/NETA	Multicenter randomized, double-blind, parallel-group, comparative trial to compare response to tibolone and raloxifene in osteopenic women
*n*	572	886	403	308
Mean age (years)	55	58 (mean years since menopause: 10)	56	66
Dose	Tibolone 2.5 mg; E2/NETA 1 mg/0.5 mg	Tibolone 2.5 mg vs. CEE/MPA 0.625/2.5 mg vs. placebo	Tibolone 2.5 mg; E2/NETA 50 μg/ 140 μg	Tibolone 1.25 mg; raloxifene 60 mg
Primary endpoint	Compare vaginal bleeding pattern between treatments	Change in mean CIMT	Changes in composite subscore for desire, arousal and satisfaction domains of the FSFI; vaginal bleeding and breast symptoms	Effects of treatment on BMD in osteopenic women
Major findings	*Bleeding*	*CIMT*	*Sexual dysfunction*	*BMD*
	Significantly less bleeding with tibolone compared with E2/NETA duringfirst 3 months (18.3% vs. 33.1%; *p* < 0.001); effect on bleeding pattern sustained throughout study	Both tibolone and CEE/MPA showed similar mean common CIMT progression that was greater than with placebo	FSFI subscores for arousal, desire, satisfaction showed a significant increase from baseline (*p* < 0.001) for both tibolone (32%) and E2/NETA (26%; per protocol analysis: *p* = 0.036 between groups)	Significant (*p* = 0.001) increases in lumbar spine BMD at week 52 for tibolone (2.2%) vs. raloxifene (1.2%; *p* < 0.01 between groups) and at week 104 (3.8% and 2.1% respectively; *p* < 0.001 between groups)
			Total FSFI score showed a significant increase from baseline (*p* < 0.001) for tibolone (31%) and E_2_/NETA (26%; per protocol analysis: *p* = 0.025 between groups)	Tibolone significantly increased hip BMD at week 52 (*p* < 0.05) and at week 104 increase was 1.26% (*p* < 0.0001) with tibolone vs. 0.44% with raloxifene (*p* < 0.05 between groups)
			FSDS showed significant reduction from baseline with both treatments (*p* < 0.001)	
	*Vasomotor*	*CVD risk factors*	*Vaginal bleeding*	
	Significant and similar reduction in prevalence of vasomotor symptoms with both treatments	Mean total cholesterol was reduced by 9.3%, 8.1% and 2.5% with tibolone, CEE/MPA and placebo, respectively	Incidence of spotting/bleeding was 16% vs. 56% (weeks 1–12) and 12% vs. 51% (weeks 13–24) for tibolone and E2/NETA, respectively (*p* < 0.001)	
		Mean HDL cholesterol decreased by 21.7% from baseline with tibolone, but increased by 9.2% and 1.2% with CEE/MPA and placebo, respectively	Vaginal hemorrhage reported as an adverse event by 11% on E2/NETA and none on tibolone (*p* < 0.001)	
	*Vaginal tissue atrophy*	*NOTE*	*Breast symptoms*	
	Similar increases for both treatments in Karyopyknotic index and Maturation index through week 48 (*p* < 0.001 vs. baseline)	Subsequent comment published that OPAL shows that neither tibolone nor CEE/MPA has beneficial effects on atherosclerosis, but does not convincingly show harm^39^	Overall incidence of breast signs and symptoms was 4% with tibolone vs. 11% with E2/NETA (*p* < 0.015 between groups)	
	*Sexuality and quality of life*			
	Greater score on McCoy's Sexuality Questionnaire with tibolone (*p* < 0.05 between groups); sexual interest significantly improved with tibolone vs. E2/NETA (*p* = 0.003 by week 48); treatments comparable in terms of effect on health-related quality of life			
	*Breast tenderness*			
	Significantly less frequently reported in tibolone group (3.2% vs. 9.8% with E_2_/NETA; *p* < 0.001)			

BMD, bone mineral density; CEE, continuous combined conjugated equine estrogen; MPA, medroxyprogesterone acetate; TVUS, transvaginal ultrasonography; 95% CI, 95% confidence interval; E2/NETA, estradiol plus norethisterone acetate; CIMT, common carotid intima media thickness; FSFI, Female Sexual Functioning Index; CVD, cardiovascular disease; HDL, high density lipoprotein; FSDS, Female Sexual Distress Scale
